# The role of interferon regulatory factors in atherosclerosis: from cell type-specific mechanisms to therapeutic strategies

**DOI:** 10.3389/fcell.2026.1795987

**Published:** 2026-06-09

**Authors:** Jie Huang, Jia Wang, Hong Chen, Fuqiang Guo, Xiaofan Yuan

**Affiliations:** 1 Department of General Practice, Sichuan Provincial People’s Hospital, School of Medicine, University of Electronic Science and Technology of China, Chengdu, China; 2 Department of Neurology, Sichuan Provincial People’s Hospital, School of Medicine, University of Electronic Science and Technology of China, Chengdu, China

**Keywords:** atherosclerosis, endothelial dysfunction, immune regulation, inflammatory response, interferon regulatory factors, macrophage polarization, therapy, vascular smooth muscle cells transformation

## Abstract

Interferon regulatory factors (IRFs) constitute a family of transcription factors that orchestrate innate and adaptive immune responses, metabolic regulation, and cellular homeostasis. Emerging evidence demonstrates that IRF1–IRF9 play central immunometabolic roles in the formation, progression, and destabilization of atherosclerotic lesions. Pro-inflammatory IRFs, particularly IRF1, IRF3, IRF5, and IRF7, promote endothelial activation, M1 macrophage polarization, pyroptosis, impaired efferocytosis, and maladaptive phenotypic transformation of vascular smooth muscle cells (VSMCs), thereby amplifying vascular inflammation and leading to plaque vulnerability. In contrast, IRF2 and IRF4 exhibit anti-inflammatory and immunoregulatory properties, restraining excessive interferon signaling, supporting M2 macrophage polarization, and maintaining Treg/Th2 homeostasis. IRF8 and IRF9 display highly context- and cell-specific functions, integrating Dendritic cells (DCs) antigen presentation, T-cell activation, and VSMC remodeling with systemic metabolic cues. Therapeutically, both direct and indirect targeting strategies for IRFs are emerging, including small-molecule inhibitors, epigenetic modulators, upstream pathway blockade, microRNA-based interventions, and nutraceutical approaches. However, given their essential roles in antiviral immunity, future translational efforts must prioritize cell-and tissue-specific IRF modulation strategies to prevent systemic immunosuppression. Collectively, IRFs represent a convergent molecular axis linking immune activation, metabolic disturbance, and hemodynamic stress. A deeper understanding of IRF signaling networks, guided by single-cell multi-omics, genetic stratification, and precision delivery systems, may enable the development of immune-metabolic therapies to address residual inflammatory risk in atherosclerotic cardiovascular diseases.

## 1 Introduction

Interferon regulatory factors (IRFs, IRF1-IRF9) constitute a family of transcription factors that control diverse biological processes, including inflammatory regulation, cell differentiation, innate and adaptive immune responses (Escalante et al., 1998; Tamura et al., 2008). All members of the IRF family share a highly conserved N-terminal DNA-binding domain (DBD) of approximately 120 amino acids, characterized by a helix-turn-helix motif that recognizes the consensus sequence A/GNGAAANNGAAACT (N represents any nucleotide) (Reich et al., 1987). These motifs were initially identified in the promoters of type I interferon-responsive genes and are collectively referred to as interferon-stimulated response elements (ISREs) (Porter et al., 1988; Eroshkin and Mushegian, 1999). The C-terminal region of most IRFs contains an IRF-associated domain (IAD; absent in IRF1 and IRF2), which mediates protein-protein interactions and exhibits structural homology to the small mothers against decapentaplegic family of transcriptional regulators, albeit with lower sequence conservation (Fuji and Kimura, 1989; Ross et al., 1984). Following phosphorylation by upstream kinases, IRFs undergo conformational activation and translocate into the nucleus, where they activate transcriptional programs which are crucial for host defense, immune modulation, and maintenance of cellular homeostasis. Atherosclerosis is widely recognized as a chronic inflammatory disorder driven by lipid deposition, endothelial dysfunction, innate and adaptive immune responses (Davies et al., 1986). The earliest stages of atherosclerosis are typically triggered by hemodynamic disturbances (Quinn et al., 1987) and subendothelial accumulation of oxidized low-density lipoprotein (ox-LDL) (Mills et al., 2000), which together induce endothelial injury. Damaged endothelial cells secrete chemokines, such as monocyte chemoattractant protein-1 (MCP-1), and upregulate adhesion molecules, including vascular cell adhesion molecule-1 (VCAM-1) and intercellular adhesion molecule-1 (ICAM-1), thereby facilitating leukocyte adhesion and transmigration into the vascular intima. Infiltrated monocytes differentiate into macrophages that engulf lipids and transform into foam cells while releasing pro-inflammatory cytokines, such as tumor necrosis factor α (TNFα) and interleukin-1β (IL-1β) (Frostegård et al., 1999). Activated T lymphocytes, particularly Th1 and Th17 subsets, potentiate the inflammatory response by secretinginterferon-γ (IFN-γ) and interleukin-17 (IL-17) (Schwartz et al., 1986). In parallel, vascular smooth muscle cells (VSMCs) migrate from the media into the intima. They proliferate and synthesize extracellular matrix components to form a fibrous cap that initially stabilizes the plaque (Clarke et al., 2006). Persistent inflammatory signaling induces VSMCs apoptosis and calcification (Massberg et al., 2002), thinning the fibrous cap and leading to plaque vulnerability. Platelets adhering to the injured endothelium release growth factors, which promote VSMCs proliferation and thrombus formation (Virmani et al., 2000). These dynamic and interdependent cellular interactions collectively promote necrotic core expansion and plaque rupture, culminating in acute cardiovascular events such as myocardial infarction and stroke (Doyle et al., 2002).

Traditionally regarded as the central regulator of antiviral defense, IRFs have recently emerged as key mediators of vascular inflammation and immune-metabolic integration. They act as downstream molecules of pattern-recognition receptors, such as Toll-like receptors (TLRs), IRFs sense pathogen-associated molecular patterns (PAMPs) and damage-associated molecular patterns (DAMPs), and translate these stimuli into transcriptional responses that modulate endothelial activation, macrophage polarization, and adaptive immunity (Fujita et al., 1988). By integrating inflammatory, metabolic, and hemodynamic cues, IRFs link innate immunity to lipid metabolism and vascular homeostasis, thereby affecting plaque development, stability, and regression.

**FIGURE 1:**
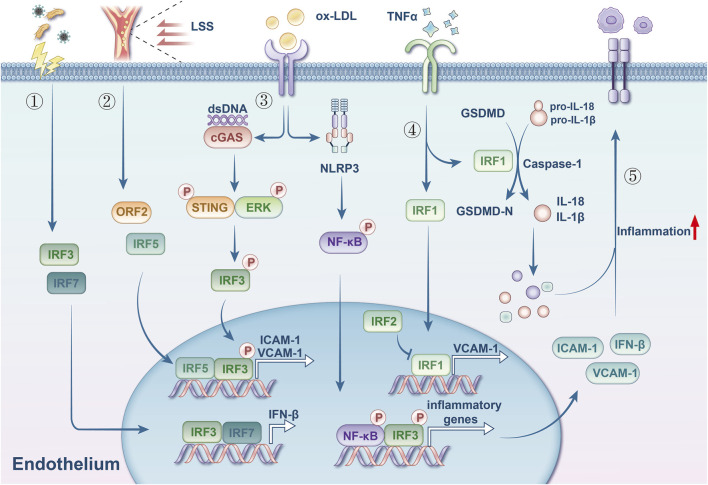
IRFs-driven endothelial activation and pyroptosis in the early stages of atherosclerosis. Under stimulation with low disturbed flow, ox-LDL, and TNFα, the endothelial cells activate the expression of IRF3, IRF5 and IRF7 (①). Phosphorylated IRF3 and IRF5 translocate into the nucleus and cooperate with NF-κB to regulate the transcription of adhesion molecules (e.g., VCAM-1, ICAM-1) and inflammatory factors such as IFN-β (②, ③). IRF1 further activates the NLRP3 inflammasome through the non-canonical NF-κB pathway, inducing caspase-1 cleavage and GSDMD-mediated pyroptosis, leading to the maturation and release of IL-18 and IL-1β (④). These inflammatory cytokines and adhesion molecules synergistically amplify endothelial inflammation, promoting monocyte adhesion and migration (⑤), thereby initiating atherosclerotic plaque formation.

**FIGURE 2:**
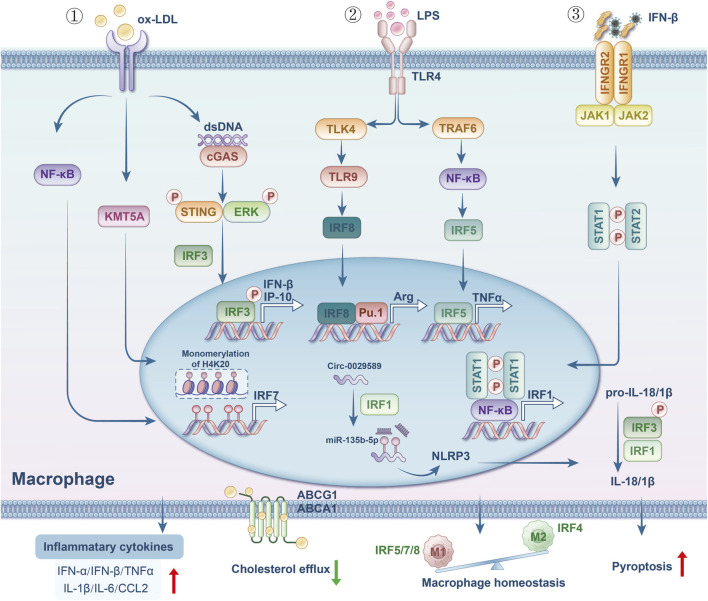
The roles of IRFs in macrophage polarization, foam cell formation, and pyroptosis. Upon ox-LDL stimulation, macrophages promote histone methyltransferase KMT5A-mediated H4K20 methylation, which cooperates with NF-κB to enhance IRF7 transcription. Concurrently, the cGAS/STING/ERK pathway facilitates IRF3 phosphorylation, leading to the induction of IFN-β and IP-10 expression (①). Under LPS stimulation, macrophages activate IRF8 via TLR4/TLR9, which cooperates with PU.1 to promote Arg transcription, while the TRAF6/NF-κB/IRF5 axis enhances TNFα transcription (②). In response to IFN-β stimulation, activation of STAT1/STAT2 synergizes with NF-κB to upregulate IRF1 expression, which in turn promotes NLRP3 inflammasome-mediated IL-18 and IL-1β production, thereby inducing macrophage pyroptosis (③). Collectively, these mechanisms amplify the expression of inflammatory cytokines, suppress ABCA1/ABCG1-mediated cholesterol efflux, promote M1 polarization (whereas IRF4 promotes M2 polarization), and enhance macrophage pyroptosis.

**FIGURE 3:**
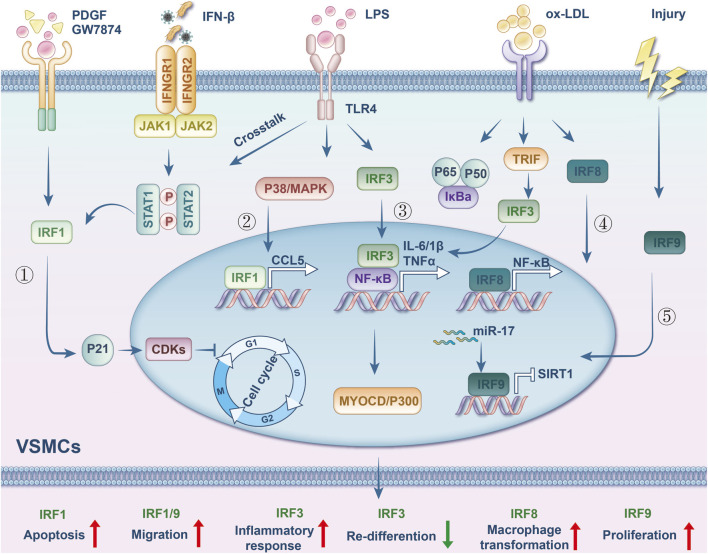
IRF-mediated regulation of phenotypic transformation, proliferation, migration, and apoptosis in VSMCs. Upon stimulation with PDGF, IFN-β, LPS, and ox-LDL, VSMCs upregulate IRF1 expression via the JAK/STAT signaling pathway, which in turn modulates VSMCs proliferation through the p21/CDKs axis (①). In response to LPS stimulation, VSMCs induce IRF1 expression via the p38/MAPK pathway, promoting the transcription of chemokines such as CCL5 and thereby enhancing cell migration (②). IRF3 is activated through the TRIF and NF-κB pathways, leading to upregulation of pro-inflammatory cytokines, including IL-6 and TNFα, which amplifies inflammatory responses and modulates the expression of cell cycle-related genes (③); MYOCD/P300 is involved in the regulation of IRF3-induced VSMCs redifferentiation. ox-LDL-induced upregulation of IRF8 in VSMCs further exacerbates these inflammatory responses (④). Under the regulation of miR-17, IRF9 modulates VSMC proliferation and phenotypic transformation via SIRT1 signaling (⑤).

**FIGURE 4:**
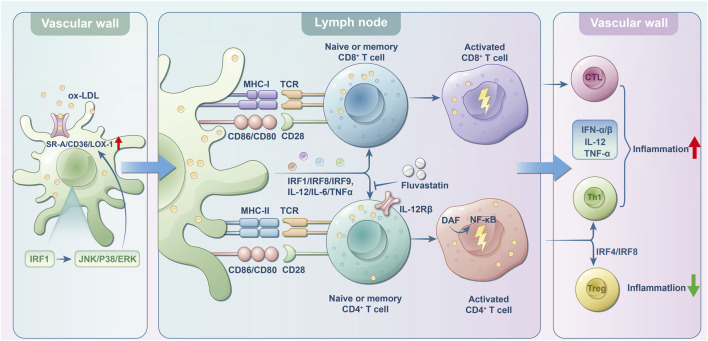
IRF-dependent regulation of DCs and T-cells in atherosclerosis. In the vascular wall, ox-LDL uptake through the scavenger receptors SR-A, CD36, and LOX-1 is enhanced in IRF1-activated antigen-presenting cells, promoting their activation and migration to lymph nodes. In lymph nodes, these cells present antigens via MHC-I or MHC-II, along with CD80/CD86 co-stimulation, to naive or memory CD8^+^ and CD4^+^ T cells. IRF1, IRF8, and IRF9 in antigen-presenting cells drive the production of IL-12, IL-6, and TNFα. These cytokines, together with IL-12Rβ-, DAF- and NF-κB-dependent signaling, promote the activation and clonal expansion of cytotoxic CD8^+^ T cells and Th1-polarized CD4^+^ T cells. Fluvastatin attenuates these IRF-dependent and cytokine-dependent priming signals. Activated CTLs and Th1 cells release IFN-α/β, IL-12, and TNF-α in the vascular wall to amplify local inflammation, whereas IRF4- and IRF8-driven Treg differentiation exerts counter-regulatory effects and limits vascular inflammation.

A comprehensive understanding of IRF-mediated signaling is essential for elucidating the molecular mechanisms that drive atherosclerosis, and for developing novel therapeutic strategies aimed at mitigating residual inflammatory risk. In this review, we provide a systematic overview of the biological functions of IRF1-IRF9, with a focus on their cell type-specific roles in endothelial activation (Figure 1), macrophage polarization (Figure 2), phenotypic switching of VSMCs (Figure 3), and adaptive immune regulation (Figure 4). In addition, we discuss emerging therapeutic approaches that target IRFs and highlight key directions for future research aimed at advancing precision immune-metabolic interventions in atherosclerotic cardiovascular diseases. By integrating mechanistic insights with translational perspectives, this review aims to offer a conceptual framework that distinguishes it from previous work (Wang et al., 2025) centered on the physiological and pathological roles of IRFs.

## 2 Cell type-specific mechanisms of IRFs in atherosclerosis

### 2.1 IRF1

As a nuclear transcription factor, IRF1 was first identified and named by Taniguchi et al. (Miyamoto et al., 1988) in 1988. It was found to mediate virus-induced activation of interferon and major histocompatibility complex (MHC) class I genes. Subsequent studies have established IRF1 as a pivotal regulator of inflammatory responses in several cell types, including endothelial cells, macrophages, VSMCs, and T lymphocytes. Through the transcriptional regulation of adhesion molecules, pyroptosis, and immune cell differentiation, IRF1 contributes to the initiation and destabilization of atherosclerotic plaques.

#### 2.1.1 Endothelial cells

Clinical studies have shown that IRF1 expression is markedly increased in patients with acute coronary syndrome (ACS) (Guo et al., 2015). Subsequent mechanistic investigations have implicated IRF1 in the regulation of monocyte-endothelial adhesion. *In vitro* overexpression studies demonstrated that IRF1 can promote monocyte adhesion to endothelial cells, partly by binding directly to the VCAM-1 promoter and enhancing its transcriptional activity following exposure to TNFα or ox-LDL (Nizamutdi et al., 2012; DeVerse et al., 2013; Qian et al., 2024). This pro-adhesive effect is potentiated by disturbed shear stress, which synergizes with hyperlipidemia to activate the IRF1-VCAM-1 axis, particularly in areas like vascular bifurcations (DeVerse et al., 2013). While monocyte adhesion is a complex process governed by multiple inflammatory pathways (including NF-κB and cytokines), these findings identify IRF1 as a contributing transcriptional regulator of this early atherogenic step.

Beyond adhesion molecule regulation, evidence from both *in vitro* and *in vivo* genetic models have expanded the role of IRF1 to include the promotion of endothelial pyroptosis. Studies using gain- and loss-of-function approaches showed that IRF1 activates the noncanonical NF-κB pathway, leading to caspase-1-dependent cleavage of gasdermin D (GSDMD) and subsequent release of IL-1β (Fan et al., 2023; Du et al., 2019). In the context of diabetic vasculopathy, hyperglycemia induces reactive oxygen species (ROS)-dependent activation of the IRF1-NLRP3 inflammasome axis, further amplifying endothelial pyroptosis and disrupting vascular barrier integrity, as demonstrated in hyperglycemic mouse models (Zhou et al., 2025). Furthermore, 25-hydroxyvitamin D has been shown to activate the IRF1/JNK/ERK/NF-κB pathway, stimulating IL-8 transcription and exacerbating inflammation-induced endothelial dysfunction (Wang F. et al., 2012). Collectively, these findings establish IRF1 as a central molecular link between metabolic stress and vascular inflammation.

#### 2.1.2 Macrophages

Proteomic profiling revealed IRF1 as a master regulator of macrophage polarization toward the pro-inflammatory M1 phenotype (Huang et al., 2018). Mechanistically, IRF1 upregulates circular RNA and promotes its m6A methylation, thereby activating the NLRP3 inflammasome and inducing caspase-1-dependent pyroptosis (Du et al., 2019; Guo et al., 2020). Additionally, the nuclear translocation of IRF1 enhances foam cell formation by upregulating scavenger receptor A (SR-A), a key mediator of cholesterol uptake and lipid accumulation (Guo et al., 2019). Through these mechanisms, IRF1 amplifies inflammatory signaling and metabolic dysregulation in macrophages, promoting the formation of necrotic cores in atherosclerotic plaques. The central role of IRF1 in coordinating macrophage activation highlights its potential as a promising therapeutic target for modulating vascular inflammation and plaque stability.

#### 2.1.3 VSMCs

In VSMCs, IRF1 mediates PPARγ-induced apoptosis and may affect the composition and stability of atherosclerotic plaques (Lin et al., 2004). Genome-wide analyses have identified a STAT1-dependent gene network co-regulated by IFN-γ and TLR4 signaling, where IRF1 cooperates with STAT1 at promoter regions to establish a pro-atherogenic transcriptional signature (Chmielewski et al., 2014). Following vascular injury, IRF1 exerts dual effects on VSMCs’ behavior. On one hand, it promotes the expression of chemokines, such as CCL5, thereby enhancing VSMCs migration and neointimal hyperplasia. Inhibition of the MAPK signaling pathway suppresses CCL5 expression, mitigating IRF1-driven neointima formation (Liu et al., 2012). On the other hand, IRF1 upregulates p21 and stimulates nitric oxide production, which can attenuate endothelial dysfunction and excessive VSMCs proliferation (Wessely et al., 2003). These findings indicate that IRF1 exerts context-dependent roles in VSMCs, balancing apoptosis, proliferation, and migration to modulate plaque stability.

#### 2.1.4 T cells

In patients with ACS, elevated levels of IRF1 contribute to T cell-driven immune dysregulation. IRF1 modulates DCs function by regulating the expression of scavenger receptors (CD36, SR-A, and LOX-1) via the JNK/P38/ERK signaling cascades, thereby affecting ox-LDL uptake and antigen presentation. It also upregulates the expression of pro-inflammatory cytokines, such as IL-12, IL-6, and TNFα, thereby enhancing DCs maturation and promoting Th1 differentiation. Simultaneously, IRF1 suppresses Treg activities, resulting in an imbalance between pro-inflammatory and anti-inflammatory immune responses (Guo et al., 2015; Guo et al., 2010). This Th1/Treg disequilibrium amplifies vascular inflammation and accelerates plaque progression. Thus, IRF1 functions as a critical immune checkpoint that integrates DCs activation with T cell-mediated inflammation during atherosclerosis.

### 2.2 IRF2

IRF2 shares substantial structural homology with IRF1 but primarily functions as its transcriptional antagonist. It competes with IRF1 for binding to the same ISREs within the promoters of type I and type II interferon-inducible genes (Fuji and Kimura, 1989). Through this competitive mechanism, IRF2 interferes with the assembly of pro-inflammatory transcriptional complexes, thereby suppressing the expression of IRF1-driven target genes, such as STAT1 (Antonczyk et al., 2025) and cathepsin S^37^. Although direct experimental evidence linking IRF2 to atherosclerosis remains limited, emerging studies from vascular and neuroinflammatory models suggest a protective and anti-inflammatory role. Notably, deletion of IRF2BP2, a transcriptional co-repressor of IRF2, in microglia overactivated NF-κB and STAT signaling pathways, exacerbating post-ischemic inflammation and tissue injury (Cruz et al., 2017). Given the shared inflammatory signaling network between cerebrovascular and atherosclerotic diseases, these findings imply that IRF2 may act as a molecular brake in the interferon signaling cascade, mitigating excessive immune activation and maintaining vascular homeostasis.

Collectively, IRF2 exerts counter-regulatory effects against pro-inflammatory IRFs, particularly IRF1, by fine-tuning the balance between interferon signaling and inflammatory gene transcription. This inhibitory control positions IRF2 as a potential anti-inflammatory modulator in the complex immune network of atherosclerosis. However, it is crucial to emphasize that direct functional evidence linking IRF2 to atherogenesis is currently lacking. Its protective role is largely inferred from its antagonistic relationship with IRF1 and findings from neuroinflammatory models, highlighting a significant knowledge gap that warrants future investigation using atherosclerosis-prone mouse models.

### 2.3 IRF3

IRF3 was initially identified as a transcription factor binding specifically to ISRE motifs and activating type I interferon genes (Au et al., 1995). It is a pivotal regulator of innate immune signaling, typically existing in an inactive cytoplasmic form. Exposure to PAMPs and DAMPs leads to IRF3 phosphorylation, dimerization, and nuclear translocation. In atherosclerosis, aberrant IRF3 activation has emerged as a molecular bridge connecting lipid metabolism, endoplasmic reticulum stress, mitochondrial dysfunction, and vascular inflammation.

#### 2.3.1 Endothelial cells

In endothelial cells, IRF3 integrates hemodynamic and metabolic stress signals that drive vascular inflammation in atherosclerosis. Disturbed shear stress activates AKT-dependent phosphorylation of IRF3 (Lv et al., 2022), while metabolic stress and endoplasmic reticulum dysfunction act through a noncanonical STING/ERK pathway to activate IRF3 (Li et al., 2023). The activated transcription factor translocates into the nucleus via KPNA2 and cooperates with NF-κB (p65) to promote the expression of type I interferons (e.g., IFN-β) (Xing et al., 2023). This sustained inflammatory signaling recruits monocytes, macrophages, and T cells into the vascular wall, perpetuating the chronic inflammation in atherosclerosis.

#### 2.3.2 Macrophages

Recent findings have shown that IRF3 is a pivotal mediator linking lipid metabolism, mitochondrial stress, and pyroptosis in macrophages during atherosclerosis. Tzieply et al. (2012) reported that ox-LDL suppresses LPS-induced IFN-β expression by inhibiting IRF3 activation. Mechanistically, this occurs through a Pellino3-and IRAK1/4-dependent modification of the adaptor protein TANK, which interferes with IRF3 phosphorylation and nuclear translocation. This phenomenon, reminiscent of viral immune evasion, suggests that ox-LDL may impede IRF3-mediated type I interferon responses, thereby reprogramming macrophage inflammatory phenotypes toward plaque instability. Fan et al. (2024) reported the central role of IRF3 in mitochondrial stress responses in atherosclerotic macrophages. Mitochondrial dysfunction leads to the release of mitochondrial DNA (mtDNA) into the cytoplasm, which activates the cGAS-STING innate immune pathway, robustly activating both IRF3 and NF-κB. This activation not only enhances the production of pro-inflammatory cytokines but also triggers GSDMD-mediated pyroptosis, establishing a pathogenic crosstalk between lipid metabolism and inflammation. Moreover, pyroptotic macrophages release mtDNA-containing microparticles, which are subsequently phagocytosed by neighboring macrophages. This uptake reactivates the STING/IRF3 pathway, forming a feed-forward cycle that sustains inflammation and pyroptosis in the plaque microenvironment. Abnormal cholesterol metabolism is also closely associated with IRF3 activation. Activation of the TLR2/4-IRF3 pathway suppresses liver X receptor and its downstream target ABCA1, thereby impairing cholesterol efflux and accelerating the formation of foam cells (Castrillo et al., 2003). These results highlight a shared molecular mechanism through which bacterial and viral signals modulate macrophage and lipid metabolism in cardiovascular health. In addition, autoimmune mechanisms may overactivate IRF3 in metabolic stress. Elevated levels of anti-dsDNA antibody have been observed in ApoE^−/−^ mice, suggesting that oxidative stress-induced DNA damage may provide endogenous ligands that engage IRF3 and perpetuate vascular inflammation (Wang Y. et al., 2012). Supporting this notion, Liu et al. (2017) also showed that genetic deletion of IRF3 in ApoE^−/−^ mice can markedly alleviate atherosclerotic lesions. This protective effect was associated with reduced macrophage infiltration, decreased expression of pro-inflammatory cytokines, and inhibited the formation of foam cells.

#### 2.3.3 VSMCs

The phenotypic switch of VSMCs from a contractile to a synthetic state, accompanied by enhanced abilities of proliferation, migration, and premature senescence, is a hallmark of atherosclerotic lesion development and progression. A growing body of evidence indicates that inflammatory stimuli, such as C-reactive protein (CRP) (Liu et al., 2010) and LPS (Ji et al., 2010), promote inflammatory activation of VSMCs via the TLR4/IRF3/NF-κB signaling pathway, producing large amounts of interferon-γ-inducible protein 10 (IP-10/CXCL10). IP-10 functions as a potent chemoattractant for activated T lymphocytes and monocytes, thereby exacerbating the inflammatory cascade in the vascular wall. Thus, the IRF3-mediated TLR4/IP-10 axis represents a critical molecular bridge linking innate immune activation to vascular remodeling. Importantly, pharmacological activation of peroxisome proliferator-activated receptor-γ (PPARγ) has been shown to inhibit this pathway (Ji et al., 2011), reducing IP-10 production and mitigating vascular inflammation, suggesting that modulation of the IRF3 signaling can serve as a potential therapeutic strategy in atherosclerosis. Beyond its role in inflammation, sustained activation of IRF3 contributes to the phenotypic transformation and premature senescence of VSMCs. Kaistha et al. (2025) reported that premature senescence fosters VSMC dedifferentiation. As a central inflammatory signaling hub, chronic IRF3 activation likely perpetuates a pro-inflammatory microenvironment that indirectly drives these phenotypic changes. This process promotes both cellular aging and maladaptive remodeling in the arterial wall.

### 2.4 IRF4

Distinct from other members of the IRF family, IRF4 can be directly activated via antigen receptor-mediated signaling and is not controlled by interferons (Matsuyama et al., 1995). A cross-ethnic genetic study identified IRF4 as a shared susceptibility gene for coronary artery disease among European, Asian, and North American populations, irrespective of ethnicity or differences in lifestyle (Gong et al., 2015). This finding highlights the universal role of IRF4 in the pathogenesis of atherosclerosis and suggests that its regulatory network may constitute a common molecular foundation for the disease across different populations.

Despite this genetic association, the mechanistic evidence for IRF4 in atherosclerosis is largely indirect. The following subsections summarize available data, but it should be noted that direct functional validation in atherosclerosis-prone mouse models is still lacking for many of these pathways.

#### 2.4.1 Macrophages

The immunity-related GTPase family M member 1 (Irgm1) has been shown to promote M1 macrophage polarization and aggravate lipid accumulation and vascular inflammation in atherosclerosis (Fang et al., 2016) Mechanistically, haploinsufficiency of Irgm1 significantly reduces the expression of the pro-inflammatory transcription factors IRF5 and IRF8, thereby limiting M1 polarization, whereas it does not affect the expression of the M2-associated transcription factor IRF4. These findings indicate that Irgm1 acts upstream of IRF5 and IRF8 to drive M1 macrophage programs. In contrast, IRF4 is a well-established master regulator of M2 macrophage polarization and anti-inflammatory responses. Although a direct regulatory interaction between IRF4 and Irgm1 has not been demonstrated in atherosclerosis models, their opposing effects on macrophage phenotype underscore the complex network of IRF family members that collectively governs vascular inflammation and plaque stability. Further studies are required to elucidate whether IRF4 and Irgm1 functionally converge on shared downstream pathways in the atherosclerotic microenvironment.

#### 2.4.2 T cells

Under hypercholesterolemic conditions, complement-mediated T cell overactivation represents a key mechanism driving the progression of atherosclerosis. Fluvastatin, through its pleiotropic anti-inflammatory properties, can suppress this process and inhibit T cell-derived cytokine secretion (Ny et al., 2021). As a master regulator of T cell differentiation, IRF4 is indispensable for the development and function of Th2 and regulatory T cells, which protect against atherosclerosis. IRF4 preserves immune balance in the arterial microenvironment, mitigating chronic inflammation and enhancing plaque stability.

#### 2.4.3 Involvement in the skeletal muscle–liver metabolic axis

Recent studies have uncovered the novel role of IRF4 beyond immune regulation, positioning it as a central mediator of inter-organ metabolic communication. In skeletal muscle, IRF4 regulates the expression of follistatin-like protein 1 (FSTL1), which activates hepatic DIP2A/CD14 signaling, thereby mediating the muscle-liver metabolic axis (Guo et al., 2023). Dysregulation of this pathway contributes to abnormal lipid metabolism in the liver and systemic inflammation, particularly in non-alcoholic steatohepatitis (NASH). Therefore, maintaining a normal IRF4/FSTL1 signaling axis in skeletal muscle and liver may indirectly protect against atherosclerosis by improving systemic metabolism. This discovery extends the role of IRF4 beyond local immune regulation, revealing a metabolic dimension through which IRF4 coordinates distant organ interactions that affect cardiovascular health. Importantly, dysfunction of the IRF4-FSTL1 axis may serve as a molecular bridge linking metabolic disorders, such as NASH, to atherosclerotic cardiovascular diseases.

### 2.5 IRF5

Unlike IRF2 and IRF4, which protect against atherosclerosis, IRF5 is a potent pro-inflammatory transcription factor that promotes plaque instability and rupture (Barnes et al., 2001). Acting as a core regulator of innate immunity, IRF5 mediates macrophage polarization, impairs efferocytosis, and accelerates fibrous cap degradation, which facilitates the progression of atherosclerotic lesions.

#### 2.5.1 Endothelial cells

Low shear stress induces epigenetic reprogramming that upregulates IRF5 expression in endothelial cells. Activated IRF5 interacts with the Aff3ir-ORF2 to drive endothelial inflammation, promoting disturbed-flow-dependent plaque formation (He et al., 2025). In contrast, deficiency of Aff3ir-ORF2 or IRF5 knockdown effectively attenuates this phenotype, suggesting that IRF5 acts as a mechanosensitive pro-inflammatory effector linking hemodynamic stress to endothelial dysfunction.

#### 2.5.2 Macrophages

IRF5 is a master transcription factor driving macrophage polarization toward the M1 phenotype. CD147 signaling through the TRAF6/IKKα axis activates IRF5, which binds to the promoter of M1 phenotype-related genes (TNF and Il12b) and enhances their transcription (Lv et al., 2024). Under disturbed flow, H3K27ac-mediated chromatin opening enhances IRF5 activation, leading to synergistic cooperation with NF-κB and promoting M1 polarization (Seneviratne et al., 2015). These macrophages release TNFα, IL-1β, IL-6, and IL-12, establishing a highly inflammatory microenvironment in atherosclerotic plaques. In addition, IRF5 disrupts efferocytosis by downregulating the expression of phagocytic receptors, such as MerTK, and interfering with phagosome maturation (Edsfeldt et al., 2022). This impairment results in the accumulation of apoptotic cells, expansion of the necrotic core, and thinning of the fibrous cap, all hallmarks of plaque vulnerability. In human coronary lesions, IRF5-expressing CD11c^+^ macrophages localize to plaque shoulders, where they secrete MMP-9 and degrade the extracellular matrix, directly contributing to plaque rupture. Although the absence of IRF5 can restore efferocytosis, reducing the area of the necrotic core by 34% (Seneviratne et al., 2017).

#### 2.5.3 Genetic and immune associations

Genetic polymorphisms in IRF5 have been linked to an increased risk of acute coronary syndromes and cardiovascular diseases (Zhang et al., 2022; Fan et al., 2010), particularly in patients with chronic inflammatory conditions, such as rheumatoid arthritis (García-Bermúdez et al., 2014) and systemic lupus erythematosus (SLE) (Watkins et al., 2015). Interestingly, although IRF5 deficiency ameliorates lupus-like systemic autoimmunity, it paradoxically accelerates high-fat-diet-induced atherosclerosis in lupus-prone models (Watkins et al., 2015). This duality suggests the potential role of IRF5 in adaptive immunity. However, no studies have directly delineated the role of IRF5 in adaptive immunity during atherosclerosis, necessitating future investigation in this regard.

### 2.6 IRF7

During the screening of ISREs-binding proteins, initially described as an ISGF3γ homolog, IRF7 was first cloned and named by Veals et al. (1993).

Independently, Zhang and Pagano (1997) identified IRF7 in Epstein-Barr virus-latently infected B cells. Recently, IRF7 has emerged as a crucial molecular hub linking innate immune activation to metabolic disturbance, with particularly prominent roles in M1 polarization of macrophages and inflammatory amplification during atherogenesis.

#### 2.6.1 Endothelial cells

Upon pathogen sensing via the mitochondrial antiviral signaling pathway, endothelial cells activate both IRF3 and IRF7, which translocate into the nucleus and jointly initiate the transcription of type I interferons, such as IFN-β, establishing a cell-autonomous antiviral state (B et al., 2010). Although these findings suggest that IRF7 may contribute to infection-associated vascular inflammation and early endothelial activation, direct evidence regarding atherosclerosis remains limited.

#### 2.6.2 Macrophages

Activation of upstream signaling pathways, including TLR9 signaling (Ma et al., 2015) and AGEs–RAGE-mediated pathways (Senatus et al., 2020; Arivazhagan et al., 2022; Ramasamy et al., 2022), induces IRF7 expression in macrophages. Elevated IRF7 expression promotes macrophage polarization toward a pro-inflammatory M1 phenotype, enhances cytokine secretion, and exacerbates inflammation. Concurrently, IRF7 suppresses the expression of ABCA1, a key regulator of cholesterol efflux, thereby impairing reverse cholesterol transport and promoting foam cell formation. IRF7^+^ macrophages are selectively recruited to vulnerable plaque regions through the CXCL10/CXCR3 chemokine axis, intensifying local inflammation (Yu et al., 2025). Additionally, histone methyltransferase KMT5A-dependent H4K20 methylation enhances the transcription of IRF7 and upregulates the production of type I interferons (IFN-α, IFN-β) and TNFα, further escalating macrophage-driven inflammation (Cheng et al., 2025).

### 2.7 IRF8

IRF8, also known as interferon consensus sequence binding protein, was first identified as an IFN-γ-inducible transcription factor (Driggers et al., 1990). IRF8 deficiency results in profound defects in myeloid development, impairing the differentiation of macrophages and DCs (Holtschke et al., 1996). In atherosclerosis, IRF8 shows highly cell-type- and context-dependent effects, forming a complex regulatory network between macrophages, VSMCs, and DCs.

#### 2.7.1 Macrophages

IRF8 participates in both pro-inflammatory and anti-inflammatory macrophage programs. The differentiation of M1 macrophage was found to be markedly impaired in hematopoietic IRF8-deficient mice, resulting in reduced atherosclerotic lesions (Döring et al., 2012). Single-cell transcriptomic studies have demonstrated that IRF8 is highly expressed in pro-inflammatory macrophages in advanced plaques, and interacts with STAT1 to integrate IFN-γ and TLR4 signals, thus amplifying the inflammatory responses (Gong et al., 2024; Chmielewski et al., 2014; Chmielewski et al., 2016). IRF8 also contributes to M2-associated transcriptional programs. Pourcet et al. (2011) showed that LXRα recruits IRF8 to the Arg1 promoter via PU.1, enhancing the expression of arginase-1 and enabling the anti-inflammatory and tissue-reparative functions of macrophages. Recent single-cell analyses have identified IRF8-associated macrophage subsets with reparative features (Gong et al., 2024). The opposing roles of IRF8 likely reflect the diversity of microenvironmental cues and signaling contexts.

#### 2.7.2 VSMCs

Single-cell atlases of atherosclerotic lesions revealed that IRF8 may function as an upstream activator of NF-κB signaling, promoting the transdifferentiation of VSMCs into macrophage-like cells and contributing to lesion complexity and instability (Gong et al., 2024).

#### 2.7.3 T cells

IRF8 plays a central role in DCs-mediated activation of T cells. In atherosclerosis, DCs process plaque-derived antigens and migrate to draining lymph nodes, where IRF8 drives antigen presentation to naive T cells and promotes differentiation into effector Th1 cells. In the study conducted by Clément et al. (Clément et al., 2018), selective deletion of IRF8-dependent DCs almost completely abolished T-cell activation and Th1 accumulation within plaques. However, IRF8 deletion also impaired Treg expansion, increasing Th1/Th17 infiltration and accelerating plaque progression. Single-cell transcriptomics also showed that IRF8 deficiency suppressed the suppressive capacity of Treg cells, potentially contributing to plaque vulnerability (Zhang et al., 2025).

### 2.8 IRF9

In 1992, Veals et al. (1992) identified IRF9 as the DNA-binding subunit of the ISGF3 complex (historically known as p48). Although structurally homologous to other IRFs, IRF9 functions strictly within the ISGF3 complex (IRF9-STAT1-STAT2) and is best known for its antiviral properties. Recent findings have indicated that IRF9 also plays crucial roles in atherosclerosis, metabolic dysfunction, and vascular repair.

#### 2.8.1 VSMCs

IRF9 is markedly upregulated following vascular injury and acts as a key driver of neointimal hyperplasia. It regulates the proliferation and migration of VSMCs through SIRT1-mediated modulation of Cyclin D1 and MMP9 expression, promoting pathological phenotypic transformation (Zhang et al., 2014). Mechanistically, the knockdown of miR-17 increases IRF9 expression, accelerating the transformation of VSMCs from a contractile phenotype to a synthetic/proliferative phenotype, a critical step in the progression of atherosclerosis and restenosis (Li et al., 2020).

#### 2.8.2 T cells

IRF9 and STAT1 have been identified as key immunologic biomarkers regulating T-cell activation during atherogenesis (Xie et al., 2024), suggesting the importance of IRF9 in bridging innate and adaptive immune responses in the vascular microenvironment.

### 2.9 Summary of cell type-specific IRF functions

The Table 1 summarizes the directionality, key targets, and evidence strength for each IRF in endothelial cells, macrophages, VSMCs, and DCs/T cells. The accompanying evidence grading framework provides a transparent rubric for evaluating the robustness of supporting data, ranging from Tier 1 (strongest) to Tier 3 (limited/indirect).

**TABLE 1:** IRF-by-cell-type functional matrix and evidence tier in atherosclerosis.

IRF	Cell type	Directionality	Key targets/Mechanisms	Evidence tier	Ref
IRF1	Endothelial cells	Pro-atherogenic	VCAM-1/GSDMD, NLRP3, NF-κB	Tier 1	21–27
	macrophages	Pro-atherogenic	NLRP3/SR-A, Caspase-1	Tier 1	25, 29, 30
	VSMCs	Context-dependent	CCL5/p21, PPARγ/STAT1	Tier 2	31–34
	DCs/T cells	Pro-atherogenic	IL-12/6/TNFα, JNK/P38/ERK	Tier 2	20, 35
IRF2	macrophages	Anti-inflammatory	STAT1/Cathepsin S	Tier 3	36, 37
IRF3	Endothelial cells	Pro-atherogenic	IFN-β, p65/AKT	Tier 1	40–42
	macrophages	Pro-atherogenic	IFN-β/mtDNA,cGAS-STING	Tier 1	43–47
	VSMCs	Pro-atherogenic	IP-10/CXCL10, TLR4/IRF3/NF-κB	Tier 2	48–50
IRF4	macrophages	Anti-atherogenic	IRGM1, iNOS	Tier 3	54
	T cells	Anti-atherogenic	Th2/Treg homeostasis, DAF/NF-κB	Tier 2	55
IRF5	Endothelial cells	Pro-atherogenic	Aff3ir-ORF2	Tier 1	58
	macrophages	Pro-atherogenic	TNFα/IL-1β/IL-6/MMP9, MerTK (Efferocytosis) (Lv et al., 2024; Edsfeldt et al., 2022; Seneviratne et al., 2017)	Tier 1	59, 61, 62
IRF7	macrophages	Pro-atherogenic	ABCA1/IFN-β, CXCL10/CXCR3	Tier 2	71–75
IRF8	macrophages	Context-dependent	Arg1/LXRα/STAT1, IFN-γ/TLR4	Tier 1	79–81
	VSMCs	Pro-atherogenic	NF-κB	Tier 2	79
	DCs	Pro-atherogenic	Th1/Th17 infiltration	Tier 1	83
IRF9	VSMCs	Pro-atherogenic	Cyclin D1/MMP9, SIRT1	Tier 2	86, 87
	T cells	Context-dependent	T-cell activation, STAT1	Tier 2	88

Evidence Grading Framework. Given the complexity and context-dependency of IRF biology highlighted in this review, we have adopted a three-tiered evidence grading system to assist readers in evaluating the strength of supporting data for each IRF–cell type interaction. Tier 1 (Strong Evidence): Supported by both human atherosclerotic lesion analysis and functional studies in mouse atherosclerosis models (e.g., ApoE^−/−^); Tier 2 (Moderate Evidence): Supported by either human genetic association studies or loss-of-function experiments in vascular injury models; Tier 3 (Limited/Indirect Evidence): Primarily based on *in vitro* cell culture studies, or extrapolation from non-cardiovascular disease models.

## 3 Therapeutic strategies targeting IRFs: current advances and future directions

The central role of IRFs in orchestrating vascular inflammation makes them promising therapeutic targets for atherosclerosis. Instead of modulating a single cytokine or lipid fraction, targeting IRFs may help reshape broader immunometabolic transcriptional programs. Current therapeutic strategies can be generally grouped into those that directly inhibit IRF activity, those that modulate their upstream regulatory networks, and emerging approaches that use epigenetic, nucleic acid, and nutraceutical interventions. Given the functional overlap and shared regulatory pathways among IRF family members, an overview organized by mechanism of action provides a clearer and more clinically relevant framework.

### 3.1 Direct pharmacological inhibition of IRFs

Directly targeting the DNA-binding or transcriptional activity of IRFs represents a logical strategy for disrupting pro-atherogenic gene programs. The novel small-molecule compound ALEKSIN was designed to bind the conserved DNA-binding domains of IRF1, IRF2, and IRF8, thereby blocking their interaction with ISREs. In ApoE^−/−^ mice, ALEKSIN treatment significantly suppressed the expression of adhesion molecules (VCAM-1, ICAM-1) and pro-inflammatory cytokines, reduced plaque burden and necrotic core size, and enhanced collagen deposition, thereby improving plaque stability (Antonczyk et al., 2025). Notably, ALEKSIN also inhibits STAT-dependent signaling, reflecting the extensive crosstalk between IRF and STAT pathways in vascular inflammation (Antonczyk et al., 2025; Sze et al., 2016). This multi-targeting effect may be therapeutically advantageous, as it simultaneously suppresses pro-inflammatory IRFs (IRF1) while modulating regulatory family members (IRF2 and IRF8).

Other pharmacological agents have demonstrated IRF-inhibitory properties. Pyrrolidine dithiocarbamate blocks IRF1 nuclear translocation and suppresses TNFα-induced VCAM-1 expression, effectively limiting monocyte-endothelial adhesion (Zhang and Frei, 2003). In vascular injury models, IRF9 knockdown suppressed VSMCs proliferation and neointimal formation, identifying IRF9 as a potential target for preventing post-intervention restenosis (Zhang et al., 2014). Myeloid cell-specific deletion of IRF5 in ApoE^−/−^ mice markedly reduced necrotic core volume by restoring efferocytosis and decreasing pro-inflammatory cytokines production (Lv et al., 2024; Seneviratne et al., 2017; Leipner et al., 2021). Furthermore, global IRF1 or IRF3 deletion in ApoE^−/−^ mice significantly attenuated atherosclerotic lesion development, primarily by suppressing endothelial pyroptosis, macrophage foam cells formation, and vascular inflammation (Du et al., 2019; Liu et al., 2017). Collectively, these genetic and pharmacological studies validate multiple IRFs as viable therapeutic targets, although the transition from experimental gene-deletion techniques to clinically feasible, drug-based inhibition methods remains a significant barrier to progress.

### 3.2 Upstream pathway modulation

Targeting upstream regulators of IRF activation offers an alternative strategy that may avoid the risks linked to direct IRF inhibition, especially considering their essential roles in antiviral immunity. These upstream nodes can be broadly grouped into cytokine signaling pathways, innate immune sensors, and metabolic or mechanical stress pathways, all of which converge on IRF activation.

Cytokine signaling pathways represent a major class of upstream regulators. The JAK-STAT axis serves as a critical node for integrating inflammatory signals. The transcriptional co-regulator CITED2 competitively binds to STAT1, thereby preventing STAT1-IRF1 complex formation and reducing the expression of downstream proinflammatory genes (Zafar et al., 2021). Similarly, IL-33 negatively regulates IRF1 via ERK1/2 activation, leading to inhibition of VCAM-1-mediated monocyte adhesion (Qian et al., 2024). The STAT1/IRF8 axis, discussed earlier as a central amplifier of vascular inflammation, has also attracted interest for its diagnostic and therapeutic potential (Chmielewski et al., 2016; Sze et al., 2016). Notably, statins, widely used lipid-lowering agents, exert pleiotropic anti-inflammatory effects in part by modulating these STAT-IRF axes. Pravastatin interferes with IFN-γ-induced IRF1 activation by altering STAT1 phosphorylation (Zhou et al., 2009), while fluvastatin suppresses complement-mediated T-cell activation, indirectly affecting IRF5 signaling (Ny et al., 2021).

Innate immune sensing pathways have emerged as critical upstream activators of IRF3 and IRF7. The cGAS-STING pathway, activated by mtDNA release during mitochondrial dysfunction, robustly activates both IRF3 and NF-κB, triggering GSDMD-mediated pyroptosis (Fan et al., 2024; Sakai et al., 2023). Pharmacological inhibition of this pathway using STING inhibitors, such as H-151 and C-176, effectively blocks IRF3 activation, suppresses vascular inflammation, and prevents plaque progression in preclinical models (Fan et al., 2024). Furthermore, the non-canonical STING/PERK pathway integrates IRF3 and NF-κB activities, leading to persistent endothelial dysfunction through epigenetic reprogramming (Li et al., 2023). This finding suggests that dual inhibitors targeting the IRF3/NF-κB transcriptional complex may offer superior efficacy compared to single-pathway blockers. TLR signaling represents another key innate immune node. Fenofibrate (Ji et al., 2010) and rosiglitazone (Ji et al., 2011) attenuate VSMCs inflammation by interfering with the TLR4/TRIF/IRF3 axis, thereby reducing IP-10/CXCL10 production. Additionally, KPNA2, a nuclear transport protein critical for IRF3 and p65 translocation, has emerged as a novel therapeutic target (Xing et al., 2023).

Metabolic and mechanical stress pathways also converge on IRFs activation. The RAGE-DIAPH1 axis is a critical upstream activator of IRF7, particularly under diabetic conditions. Inhibitors such as soluble RAGE and DIAPH1 antagonists effectively block hyperglycemia-mediated IRF7 activation (Senatus et al., 2020; Arivazhagan et al., 2022; Ramasamy et al., 2022). CD147 drives M1 macrophage polarization and impairs efferocytosis partly through IRF5 (Lv et al., 2024), while the newly characterized nested gene Aff3ir mediates disturbed-flow-induced IRF5 activation (He et al., 2025). Together, these upstream nodes highlight the therapeutic potential of intercepting the diverse mechanical, metabolic, and immune cues that converge on IRF signaling. By targeting these nodes, it may be possible to modulate IRF activities in a context-dependent manner, potentially achieving greater specificity than direct IRF inhibition.

### 3.3 Epigenetic regulation of IRFs

Recent evidence has expanded the regulatory landscape of IRFs from traditional transcriptional control to include epigenetic modulation. This shift is important for understanding how environmental and metabolic stimuli, such as disturbed flow, hyperglycemia, and oxidative stress, can cause lasting changes in IRF activity and vascular cell function without altering the DNA sequence. Epigenetic mechanisms provide a framework for this kind of persistent and context-dependent gene regulation. They act at several interconnected levels, including histone modification, chromatin remodeling, and RNA methylation.

At the level of histone modification, methylation serves as a key determinant of IRFs gene expression. KMT5A-dependent H4K20 methylation has been identified as a critical epigenetic mark that enhances IRF7 transcription and promotes type I interferon production in macrophages, thereby amplifying inflammatory responses (Cheng et al., 2025). The therapeutic relevance of targeting this pathway is underscored by the finding that the traditional Chinese medicine formulation Shexiang Baoxin Pill inhibits KMT5A-mediated H4K20 methylation and downregulates IRF7 transcription, demonstrating the feasibility of pharmacological intervention at the epigenetic level (Cheng et al., 2025).

Beyond local histone marks, chromatin remodeling controls how accessible IRF target genes are to the transcriptional machinery. This mechanism is especially important in translating mechanical forces into sustained inflammatory phenotypes. Under disturbed flow conditions, H3K27ac-mediated chromatin opening increases the accessibility of IRF5 target loci. This facilitates IRF5 binding and promotes M1 macrophage polarization (He et al., 2025; Seneviratne et al., 2015). These findings help explain how hemodynamic stress can drive stable pro-atherogenic transcriptional programs through epigenetic reprogramming.

At the post-transcriptional level, RNA methylation has emerged as a novel epigenetic layer controlling IRF-driven inflammation. IRF1 promotes m6A methylation of specific circular RNAs, which in turn activates the NLRP3 inflammasome and induces caspase-1-dependent pyroptosis in macrophages (Du et al., 2019; Guo et al., 2020). This IRF1-m6A-NLRP3 axis reveals a feed-forward regulatory circuit in which an IRF protein orchestrates its own downstream inflammatory effects through epitranscriptomic modulation.

Collectively, these interconnected epigenetic mechanisms, including histone methylation, chromatin remodeling, and RNA methylation, form a multi-layered regulatory network that shapes IRF function in atherosclerosis. Unlike genetic mutations, epigenetic modifications are reversible by nature, which makes them appealing targets for therapeutic intervention.

### 3.4 Nucleic acid-based and gene silencing approaches

Advances in nucleic acid therapeutics have made it possible to develop strategies that selectively regulate IRF expression. MicroRNA-based approaches have shown strong potential. miR-22 shows anti-atherosclerotic effects by directly suppressing IRF5 expression (Wu et al., 2021), which suggests that restoring or mimicking miR-22 activity could be a useful therapeutic option. In a similar way, miR-17 negatively regulates IRF9, and restoring miR-17 levels may help prevent pathological phenotypic changes in VSMCs (Li et al., 2020). Gene silencing strategies have also confirmed several IRFs as therapeutic targets. For example, IRF9 knockdown effectively reduces VSMCs proliferation and neointimal formation after vascular injury (Zhang et al., 2014), while CXCR3 antagonists block the recruitment of IRF7-positive macrophages to necrotic cores (Yu et al., 2025).

However, systemic inhibition of IRFs remains a major challenge because of their key roles in antiviral defense and immune surveillance. This issue is further complicated by the cell-type-specific functions of IRFs in the vascular wall. In DCs, inhibiting IRF1 reduces Th1-driven inflammatory responses (Guo et al., 2015; Guo et al., 2010). In macrophages, targeting IRF1-dependent m6A modification may help prevent pyroptosis (Guo et al., 2020; Guo et al., 2019). In VSMCs, modulating IRF1-related signaling can limit maladaptive remodeling (Lin et al., 2004; Liu et al., 2012). These findings highlight the need for more precise strategies that can achieve cell-type-specific regulation of IRF activities.

Consequently, developing vascular-targeted delivery systems is essential to achieve localized IRF modulation while minimizing systemic immunosuppression (Sze et al., 2016). Approaches such as nanoparticle-based carriers, antibody-conjugated inhibitors, and cell-type-specific RNA therapeutics are being explored. Improving precision delivery remains a key challenge for translating these findings into clinical use.

### 3.5 Nutraceutical and natural compound interventions

Bioactive natural compounds have gained attention as supportive strategies for regulating IRF signaling in atherosclerosis. They offer advantages such as low toxicity and the ability to act on multiple targets. However, most of the current evidence comes from preclinical studies, and the underlying mechanisms are often not fully clear.

These compounds can be grouped based on their main modes of action. Some act on upstream kinase pathways. For example, berberine reduces endothelial inflammation by lowering AKT and IRF3 phosphorylation (Lv et al., 2022). Others influence IRF transcriptional activity either directly or indirectly. Sea buckthorn extract inhibits IRF1 while increasing antioxidant enzyme activity (Padmavathi et al., 2005), trans-caryophyllene reduces IRF1-mediated leukocyte adhesion (Zhang et al., 2017), and Aronia berry extract downregulates IRF1 through STAT3 modulation (Iwashima et al., 2019). A third group works through metabolic or epigenetic regulators. Flavonoids influence IRF5-related interferon signaling (Ruskovska et al., 2024), the ApoA-I mimetic peptide 4F reduces IRF5 expression (Xu et al., 2012), and LXR agonists inhibit the TLR9/IRF7 pathway, which reduces foam cell formation (Sorrentino et al., 2010).

Despite these encouraging results, several limitations preclude their current clinical translation. Mechanistic studies often remain at a descriptive level, and many compounds, such as berberine, face fundamental pharmacokinetic hurdles including extremely poor oral bioavailability, which limits *in vivo* efficacy (Table 2). Furthermore, the evidence for clinical benefit in cardiovascular disease is largely restricted to association studies rather than interventional trials. Therefore, while nutraceuticals represent a promising adjunctive approach, their development as *bona fide* IRF-targeted therapies requires rigorous optimization of bioavailability and validation in large-scale clinical studies.

**TABLE 2:** Therapeutic strategies targeting IRF signaling in atherosclerosis.

Therapeutic strategies	Target	Agent	Stage of evidence	Key translational hurdle	Primary safety concern	Ref
Direct inhibitors	IRF1/2/8	ALEKSIN	ApoE^−/−^ Mouse	Disrupt IRF2-mediated homeostasis	Compromised antiviral defense	36, 89
	IRF1	Pyrrolidine dithiocarbamate	*In vitro*	Bioavailability and stability *in vivo*	Metal chelation off-target effects	90
Upstream pathway modulators	IRF3	STING inhibitors	ApoE^−/−^ Mouse	Translating acute inflammation to chronic disease	Increased susceptibility to virus infection	44
	IRF7	RAGE/DIAPH1 antagonists	Diabetic Mouse	Specificity for diabetic atherosclerosis	Off-target effects on axonal growth	71–73
Epigenetic modifiers	IRF7	Shexiang Baoxin pill	Mouse/*In vitro*	Bioavailability of complex natural mixtures	Off-target histone methyltransferase effects	75
Nucleic acid/Gene silencing	IRF5	miR-22 mimics	*In vitro*/Mouse	Efficient vascular-targeted delivery	Off-target mRNA silencing in liver	95
	IRF9	miR-17 restoration	*In vitro*	Endosomal escape of RNA therapeutics	Immunostimulatory RNA sensor activation	87
Nutraceuticals	IRF3	Berberine	*In vitro*/Mouse	Extremely low oral bioavailability	Interaction with CYP450 enzymes	40
	IRF5	Flavonoids	Human Association	Gut microbiota metabolism variability	Generally low	99

### 3.6 Consensus, controversies, and future directions

There is broad agreement that IRFs, especially IRF1, IRF3, IRF5, and IRF8, act as key transcription factors driving vascular inflammation and are promising therapeutic targets. A large body of preclinical evidence shows that genetic or pharmacological inhibition of these factors can reduce the development of atherosclerotic lesions and promote plaque stabilization. Among these findings, the identification of the cGAS-STING-IRF3 axis has been particularly important. It establishes a central link between mitochondrial stress, pyroptosis, and vascular inflammation (Fan et al., 2024; Sakai et al., 2023). These results provide a solid basis for developing IRF-targeted anti-inflammatory therapies. However, the complexity of IRF biology creates clear challenges for therapeutic use. A major issue comes from the dual and context-dependent roles of certain IRFs. For instance, IRF5 deficiency exerts divergent effects depending on disease context—ameliorating lupus while exacerbating atherosclerosis in lupus-prone mice (see Section 2.5.3) (Watkins et al., 2015). Similarly, BAFF neutralization unexpectedly worsens disease progression by suppressing plasmacytoid DCs function and weakening IRF7 signaling (Tsiantoulas et al., 2018). These findings show that IRFs can play opposite roles depending on the disease setting and cell type. In addition, IRF8 and IRF9 display highly flexible and sometimes conflicting functions across immune and vascular cells (Döring et al., 2012; Gong et al., 2024; Zhang et al., 2014; Wang et al., 2013). Although broad-spectrum IRF inhibitors such as ALEKSIN show beneficial effects, they may disrupt the balance between pro-inflammatory and regulatory IRFs and could lead to unintended immunosuppressive risks. Therefore, the key challenge is how to precisely control specific IRF functions within a complex signaling network.

To address these issues and advance IRF-based therapies toward clinical application, future research should focus on several directions. First, the cell-specific roles of IRFs need to be clarified. Their functions in different cell types, including endothelial cells, macrophages, VSMCs, and T cells, as well as at different stages of disease progression, are still not fully understood. Using single-cell multi-omics approaches and cell-specific gene editing animal models will help map how IRFs function in different contexts and support more precise interventions. Second, integrating human genetic data is important for patient stratification. The IRF family shows many genetic polymorphisms, which may affect how patients respond to treatment. Combining human genetic data with IRF polymorphism information can help identify the patients who are most likely to benefit and support personalized treatment strategies. Third, more attention should be given to the metabolic roles of IRFs. Their functions go beyond immune regulation, and they are involved in inter-organ metabolic communication. One example is the IRF4-FSTL1-mediated muscle-liver axis (Guo et al., 2023), which links systemic metabolism with vascular inflammation. Understanding these metabolic roles may open new ways to design combined immune and metabolic therapies. Fourth, targeted delivery systems need to be improved. Systemic inhibition of IRFs may interfere with their essential antiviral functions, so developing cell- or tissue-specific delivery methods such as nanocarriers, antibody-drug conjugates, and cell-type-specific nucleic acid therapeutics is critical for balancing efficacy and safety (Sze et al., 2016). Finally, the timing and combination of therapies need further study. The optimal timing, duration, and combination of IRF-targeted treatments with standard lipid-lowering or anti-inflammatory therapies remain unclear, and more systematic preclinical studies are needed to inform clinical trial design.

In summary, therapies targeting IRFs offer strong potential to address the remaining inflammatory risk that persists even after effective lipid-lowering treatment. Moving beyond a purely lipid-focused view and considering integrated immune and metabolic regulation may open new directions. If the challenges discussed above can be addressed, IRFs could become important targets for next-generation therapies for atherosclerotic cardiovascular disease.

## 4 Discussion

The IRF family has become an important transcriptional hub that connects innate immunity, metabolic stress, and vascular inflammation in atherosclerosis. By bringing together cell-specific mechanisms with a closer look at emerging therapeutic strategies and future research directions, this review offers a more practical framework that goes beyond earlier summaries of IRF roles in vascular disease (Wang et al., 2025).

IRFs act as molecular integrators at the intersection of several pathogenic pathways. IRF3 is a clear example, as it works as a shared downstream effector for both the cGAS-STING DNA sensing pathway and the TLR3/4-TRIF axis. In this way, it links mitochondrial stress, pathogen sensing, and sterile inflammation to vascular dysfunction (Li et al., 2023; Fan et al., 2024; Sakai et al., 2023). IRF1 also integrates signals from IFN-γ, TNFα, and disturbed flow, coordinating endothelial activation, macrophage pyroptosis, and VSMC phenotypic switching (Nizamutdi et al., 2012; DeVerse et al., 2013; Qian et al., 2024; Fan et al., 2023; Du et al., 2019; Chmielewski et al., 2014). Because of this integrative role, IRFs serve as key points where hemodynamic, metabolic, and inflammatory signals are brought together and translated into transcriptional responses that shape disease progression. Notably, IRFs function within a tightly balanced regulatory network shaped by opposing forces. The interplay between pro-inflammatory members (IRF1, IRF3, IRF5, IRF7) and anti-inflammatory members (IRF2, IRF4) governs the overall inflammatory milieu of the vessel wall (Antonczyk et al., 2025; Storm van’s Gravesande et al., 2002; Fang et al., 2016; Ny et al., 2021). This balance changes depending on the disease context, which has clear implications for therapy. Although broad inhibition of pro-inflammatory IRFs may seem appealing, it can disrupt this balance and lead to unintended outcomes. For example, BAFF neutralization suppresses IRF7-dependent plasmacytoid dendritic cell function but unexpectedly accelerates atherosclerosis, showing that interfering with immune balance can produce the opposite effect (Tsiantoulas et al., 2018). Similarly, as noted in Section 2.5.3, IRF5 deficiency paradoxically accelerates atherosclerosis in lupus-prone models despite improving autoimmunity (Watkins et al., 2015).

The context-dependent behavior of IRFs adds further complexity. IRF8 promotes pro-inflammatory macrophage polarization in some settings, but in other cases it supports M2-related repair processes (Döring et al., 2012; Gong et al., 2024; Pourcet et al., 2011). IRF9 drives abnormal VSMC proliferation after vascular injury, yet it also protects against hepatic insulin resistance and steatosis (Zhang et al., 2014; Wang et al., 2013). These contrasting roles within the same molecule show that IRF function is shaped by interactions with cell-specific co-factors and epigenetic environments rather than being fixed. This challenges simple views that label IRFs as either beneficial or harmful and instead points to the need for approaches that fine-tune their activity rather than completely block it.

When looking at the evidence linking specific IRFs to atherosclerosis, there is clear variation in how strong the causal support is. For IRF1, IRF3, and IRF5, loss-of-function studies in ApoE^−/−^ mice provide strong evidence. These studies show reduced lesion formation, smaller necrotic core areas, and improved plaque stability when these genes are deleted (Du et al., 2019; Liu et al., 2017; Seneviratne et al., 2017; Leipner et al., 2021). These results are supported by defined mechanisms such as pyroptosis, inflammation, and efferocytosis (Du et al., 2019; Guo et al., 2020; Fan et al., 2024; Edsfeldt et al., 2022; Seneviratne et al., 2017; Sakai et al., 2023). For IRF2 and IRF4, the evidence is more indirect. It mainly comes from their opposing roles, genetic association studies, and links to M2 polarization and Treg maintenance (Cruz et al., 2017; Gong et al., 2015; Fang et al., 2016; Ny et al., 2021), and direct evidence from atherosclerosis models is still lacking. IRF7, IRF8, and IRF9 show the most complexity, with functions that depend strongly on cell type and disease stage. Studies on BAFF neutralization illustrates the peril of disrupting immune balance, as it accelerates atherosclerosis despite suppressing certain IRF7-dependent responses (Tsiantoulas et al., 2018). IRF8 shows opposite effects, promoting T-cell activation in dendritic cells while also regulating both pro- and anti-inflammatory macrophage subsets (Döring et al., 2012; Clément et al., 2018). IRF9 also has dual roles, promoting vascular remodeling but protecting against metabolic dysfunction (Zhang et al., 2014; Wang et al., 2013). Together, these findings show clear differences among IRF family members and highlight the need to consider cell type, disease stage, and tissue context when designing therapies.

Recent single-cell and spatial transcriptomic profiling of human atherosclerotic lesions has provided critical validation for IRF functions derived from murine models. Human plaque analyses have identified IRF8 as a potential driver of SMC-to-macrophage transdifferentiation (Gong et al., 2024), while IRF7 has been implicated in maladaptive SMC phenotype switching and correlates with plaque instability (Liu et al., 2025). Integrated single-cell atlases and spatial transcriptomics further delineate specialized macrophage subsets and regional transcriptional patterns within human lesions (Träuble et al., 2025; Gastanadui et al., 2024). Despite this convergence, key preclinical limitations persist. ApoE^−/−^ and Ldlr^−/−^ mice differ in lipoprotein composition and mechanisms of atherogenesis (Getz and Reardon, 2016); moreover, the influence of biological sex, a critical determinant of IRF5 activity and interferon response remains largely unexplored in the context of vascular inflammations (Griesbeck et al., 2015). A comprehensive understanding of IRF biology in atherosclerosis therefore requires deliberate integration of findings from complementary animal models and focused efforts to delineate sex-specific mechanisms. Such insights will be essential for the rational design of future therapies.

Despite these advances, several key gaps remain. The cell-specific roles of IRFs in atherosclerosis are not fully mapped, as many studies rely on global knockout models or bulk analyses that do not capture cellular diversity (Gong et al., 2024). The timing of IRF activation at different disease stages is also not well understood, making it difficult to define the best window for intervention. The metabolic roles of IRFs, such as the IRF4-FSTL1 muscle-liver axis, have not been studied in enough detail, even though atherosclerosis is increasingly seen as a disease involving combined immune and metabolic imbalance (Guo et al., 2023). Human genetic evidence linking IRF polymorphisms to cardiovascular risk is still limited and often lacks sufficient power, which makes patient stratification difficult (Gong et al., 2015; Zhang et al., 2022; Fan et al., 2010; García-Bermúdez et al., 2014). In addition, there is still a large gap between preclinical findings and clinical use. Issues such as bioavailability, target specificity, and delivery strategies remain largely unresolved (Sze et al., 2016).

Looking ahead, progress in this field will depend on moving from descriptive studies to a clearer understanding of how IRFs integrate signals over time and across different tissues. Single-cell multi-omics approaches can help define IRF functions at the cellular level and show how different cell types use these factors to drive specific responses (Gong et al., 2024). Studies that focus on different disease stages in animal models can help identify when IRF-targeted interventions are most effective. Research on the role of IRFs in inter-organ metabolic communication may reveal new therapeutic entry points (Guo et al., 2023). Large-scale genetic studies with well-characterized patient groups can help identify those most likely to benefit from IRF-targeted treatments. Finally, improving cell-type-specific delivery systems, including nanoparticles, antibody-drug conjugates, and nucleic acid therapeutics, may allow precise control of IRFs without causing broad immunosuppression, which has limited clinical translation so far.

## 5 Conclusion

Overall, the IRF family represents a crucial molecular nexus linking immune activation, metabolic dysfunction, and vascular inflammation. With advances in translational science, targeting IRFs holds great promise for developing next-generation precision therapies, particularly for patients with high inflammatory risk or insufficient response to conventional lipid-lowering agents.
